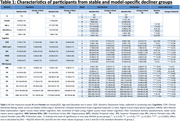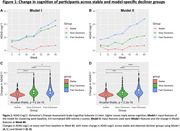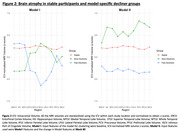# A weakly‐supervised, MRI‐guided clustering of cognitive decline: A post‐hoc analysis of the EXPEDITION‐3 clinical trial

**DOI:** 10.1002/alz.093891

**Published:** 2025-01-09

**Authors:** Bhargav Teja Nallapu, Kellen K. Petersen, Idris Demirsoy, Richard B. Lipton, Christos Davatzikos, Laura Rabin, S. Ahmad Sajjadi, Ali Ezzati

**Affiliations:** ^1^ Albert Einstein College of Medicine, Bronx, NY USA; ^2^ University of California, Irvine, Irvine, CA USA; ^3^ Perelman School of Medicine, University of Pennsylvania, Philadelphia, PA USA; ^4^ Brooklyn College of the City University of New York, Brooklyn, NY USA; ^5^ Institute for Memory Impairments and Neurological Disorders (UCI‐MIND), University of California, Irvine, Irvine, CA USA; ^6^ University of California Irvine, Irvine, CA USA

## Abstract

**Background:**

Alzheimer's disease exhibits heterogeneity through varied phenotypic and pathological manifestations. Here, we aimed to investigate the potential of semi‐supervised pattern classification applied to volumetric MRI data in identifying relatively homogeneous subgroups of individuals exhibiting cognitive decline (CD) throughout the study period.

**Method:**

We used data from the placebo arm of trial of Solanezumab for mild dementia due to AD (EXPEDITION‐3 trial). Participants were classified as showing CD if there was any increase in Clinical Dementia Rating (CDR, Sum of Boxes) from baseline to the 80 weeks of follow‐up, and as stable cognition (SC) otherwise. We applied a non‐linear, semi‐supervised clustering algorithm, HYDRA, that captures subtypes within the decliner group accounting for the heterogeneous variations that are also present in the stable group. Two models with different feature‐sets were developed: model I used only baseline volumetric MRI measures, while the model II included both baseline volumetric MRI measures and longitudinal changes observed after 80 weeks.

**Result:**

Participants were 710 individuals with longitudinal MRI and CDR measures (mean age: 72.57[±7.69] years, 60.1% Female). Models I and II each produced two distinct subgroups of CD with notable differences in their baseline MRI volumes, and different rates of change in cognitive outcomes over the course of trial (Table 1). In both the models, one of the subgroups (Slow Decliner) had significantly lower change in scores than the other (Fast Decliner) on the 11‐item version of the Alzheimer’s Disease Assessment Scale–Cognitive subscale (ADAS‐Cog) at the end of Week 80 (Figure 1). In Model‐I, both the decliner subgroups had smaller temporal and parietal lobe volumes at baseline compared to the Stable group (Figure 2). In Model‐II, the Slow Decliner group had higher baseline MRI volumes than the stable group and significantly lower change in ADAS‐Cog compared to that of the Fast Decliner group (Figure 2).

**Conclusion:**

Heterogeneity in brain atrophy is linked to distinct patterns of cognitive decline. Using longitudinal changes in MRI measures revealed decliner subgroups with greater difference in ADAS‐cog scores compared to those obtained by baseline measures only.